# Efforts to Recruit Medical Students From Rural Counties: A Model to Evaluate Recruitment Efforts

**DOI:** 10.7759/cureus.17464

**Published:** 2021-08-26

**Authors:** Gary L Beck Dallaghan, Julie C Spero, Julie S Byerley, Lisa Rahangdale, Erin P Fraher, Beat Steiner

**Affiliations:** 1 Office of Medical Education, University of North Carolina at Chapel Hill School of Medicine, Chapel Hill, USA; 2 Cecil G. Sheps Center for Health Services Research, University of North Carolina at Chapel Hill School of Medicine, Chapel Hill, USA; 3 Pediatrics, University of North Carolina at Chapel Hill School of Medicine, Chapel Hill, USA; 4 Obstetrics and Gynecology, University of North Carolina at Chapel Hill School of Medicine, Chapel Hill, USA; 5 Family Medicine, University of North Carolina School of Medicine, Chapel Hill, USA

**Keywords:** medical school, admissions, rural recruitment, pipeline programs, physician workforce

## Abstract

Background

Over the past 40 years, the physician supply of North Carolina (NC) grew faster than the total population. However, the distribution of physicians between urban and rural areas increased, with many more physicians in urban areas. In rural counties, access to care and health disparities remain concerning. As a result, the medical school implemented pipeline programs to recruit more rural students. This study investigates the results of these recruitment efforts.

Methodology

Descriptive analyses were conducted to compare the number and percentage of rural and urban students from NC who applied, interviewed, and were accepted to the University of North Carolina’s School of Medicine (UNC SOM). The likely pool of rural applicants was based on the number of college-educated 18-34-year-olds by county.

Results

Roughly 10.9% of NC’s population of college-educated 18-34-year-olds live in rural counties. Between 2017 and 2019, 9.3% (n = 225) of UNC SOM applicants were from a rural county. An increase of just 14 additional rural applicants annually would bring the proportion of rural UNC SOM applicants in alignment with the potential applicant pool in rural NC counties.

Conclusions

Our model of analysis successfully calculated the impact of recruitment efforts to achieve proportional parity in the medical school class with the rural population of the state.* *Addressing rural physician workforce needs will require multiple strategies that affect different parts of the medical education and healthcare systems, including boosting college completion rates in rural areas. This model of analysis can also be applied to other pipeline programs to document the success of the recruitment efforts.

## Introduction

Rural populations have poorer health outcomes and higher mortality rates [1], stemming from lack of health insurance coverage, health behavior and risk factors, lower socioeconomic status, and physician shortages in rural areas [2,3]. Over the past four decades, even though the physician supply of North Carolina (NC) has grown, the maldistribution of physicians in urban versus rural areas has increased [4,5]. In 1979, urban counties had about 6.1 more physicians per 10,000 population compared to rural counties. By 2018, this gap had increased to 13.1 more physicians per capita.

Unfortunately, rural students are underrepresented in medical schools nationwide and their numbers are declining [6]. Because growing up in a rural area is a strong predictor of future rural practice [7-12], recruiting rural medical school applicants is an important strategy to boost the rural physician workforce. The causes of the underrepresentation of rural students have been attributed to a shortage of applicants [13,14]. Some studies indicate rural applicants are equally likely to be admitted to medical school relative to urban applicants [6,15].

Challenges recruiting rural applicants to medical school may be explained by Bourdieu’s theory of habitus [16]. Individual’s educational paths and potential access are constrained by habitus [17], where habitus is defined as a set of dispositions, including family background, rural versus urban upbringing, and financial status [16]. In one study, children of farmers were less likely to pursue higher education than children of professionals [18]. Bourdieu’s theory underscores challenges facing rural students accessing higher education due to a lack of role models.

The University of North Carolina at Chapel Hill’s School of Medicine (UNC SOM) plays a key role in producing a physician workforce that meets the state’s healthcare needs. Due to the rising gap in rural versus urban physicians, UNC SOM established multiple pipeline programs to recruit rural students, specifically, the Middle School Science and Technology Enrichment Program (middle school), Health Professions Recruitment and Exposure Program (high school), and Medical Education Development Program (college). This study presents a model for analyzing these recruitment efforts. A preprint of this article was previously posted to Research Square on February 5, 2021.

## Materials and methods

Sample

All in-state applicants for the 2017, 2018, and 2019 admission cycles were included in the analyses (n = 2,791). A total of 350 applications (112 in 2017 and 2018, and 126 in 2019) were excluded because they were either incomplete or withdrawn. Both first-time and re-applicants were included in the sample.

Data and methods

This descriptive analysis used application data from the UNC SOM in Chapel Hill. The UNC SOM admissions data contain variables collected by the Association of American Medical Schools’ (AAMC’s) American Medical College Application Service® (AMCAS®), the centralized application service used by most allopathic medical schools in the United States.

In addition to the AMCAS application data, we used annual certified population estimates from the State Demographer’s Office at the NC Office of State Budget and Management. Rural status was assigned based on the applicant’s permanent address and was defined using the August 2017 Federal Office of Management and Budget Standards for Delineating Metropolitan, Micropolitan, and non-Core Based Statistical Areas. Metropolitan counties were considered “urban” and all other counties were considered “rural.” Using this definition, NC had 54 rural counties during the study period.

We used data from the American Community Survey to define a pool of potential applicants based on the number of college-educated 18-34-year-olds in each county. While there is no age limit for applicants, the majority (98.0%, n = 2,734/2,791) of all UNC SOM applicants during the study period were between the ages of 18 and 34. Limiting the population by age allowed us to better estimate the potential applicant pool.

Analysis

Descriptive statistics and bivariate analyses were conducted in Stata version 14 (StataCorp LP, College Station, TX). This study was approved by the UNC-Chapel Hill Office of the University Registrar, which is required to approve access to student records for research purposes. The study was reviewed by the UNC-Chapel Hill Office of Human Research Ethics and received an Internal Review Board exemption under the secondary data exemption category.

## Results

**Figure 1:**
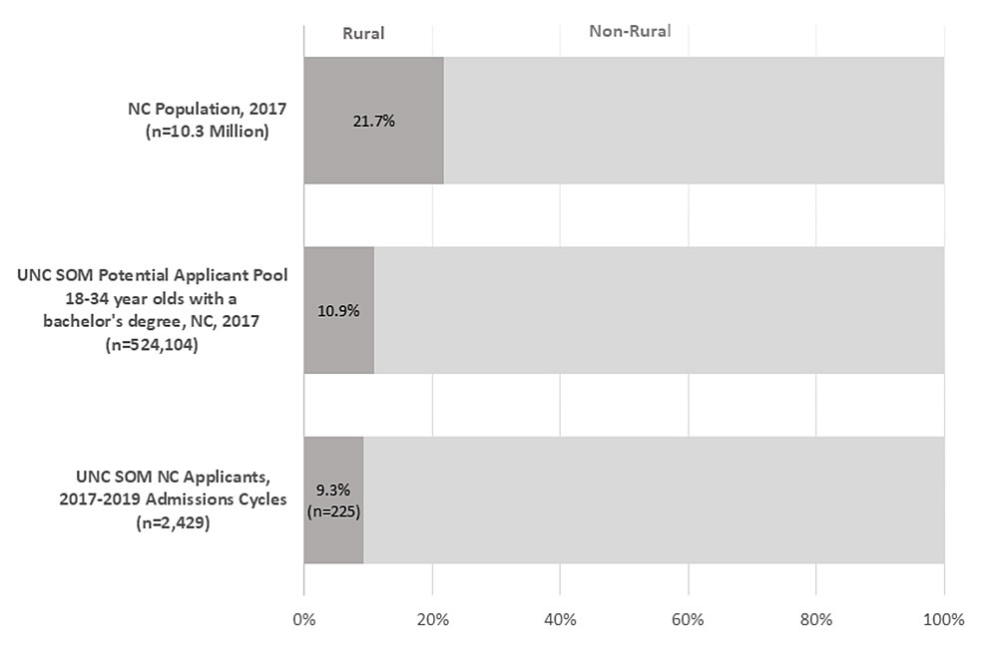
Rurality of NC’s population, potential UNC SOM applicant pool, and UNC SOM applicants. Source: Sheps Center for Health Services Research with applicant data from the School of Medicine at the University of North Carolina-Chapel Hill for the 2016-2017, 2017-2018, and 2018-2019 admission cycles. July 2017 Certified Current Population Estimates from the NC Office of State Budget and Management. Data on educational attainment by county is from the U.S. Census Bureau, 2013-2017 American Community Survey 5-Year Estimates. NC: North Carolina; UNC SOM: University of North Carolina at Chapel Hill’s School of Medicine

Although 2.2 million (21.7%) North Carolinians live in rural areas, the proportion of potential medical school applicants in rural areas is smaller, largely limited to college-educated 18-34-year-olds in each county. Figure 1 shows that roughly 10.9% of NC’s population of college-educated 18-34-year-olds live in rural NC counties [19].

Table 1 shows data on UNC medical school applicant characteristics by urban versus rural home address. Roughly half of the rural (50%, n = 130) and urban applicants (50%, n = 1,268) were interviewed, and roughly one in five applicants from each group (21%, n = 54; 20%, n = 498, respectively) were offered admission. The proportion of admitted applicants from rural NC (9.8%, n = 54/552) was similar to the proportion of all applicants from rural NC (9.2%, n = 257/2,791), and was close to the proportion of the total population of potential applicants in rural NC (10.9%). Had an additional six applicants applied each year, the proportion of rural applicants would have aligned with the potential rural applicant pool (257 + 18 = 275 or 9.8% of 2,791).

**Table 1:** Characteristics of rural versus urban UNC SOM applicants from North Carolina who applied, received an interview, and were offered admission during the 2017-2019 application cycles. Source: Sheps Center for Health Services Research with applicant data from the School of Medicine at the University of North Carolina-Chapel Hill for the 2016-2017, 2017-2018, and 2018-2019 admission cycles. UNC SOM: University of North Carolina at Chapel Hill’s School of Medicine

	Applied				Interviewed				Admitted			
	Rural		Urban		Rural		Urban		Rural		Urban	
	#	%	#	%	#	%	#	%	#	%	#	%
Total	257		2,534		130		1,268		54		498	
Application cycle												
2017	92	36%	796	31%	44	34%	416	33%	18	33%	127	26%
2018	84	33%	872	34%	42	32%	421	33%	14	26%	179	36%
2019	81	32%	866	34%	44	34%	431	34%	22	41%	192	39%
Rural birth county	78	30%	96	4%	45	35%	49	4%	22	41%	19	4%

The rural and urban in-state applicant pools had similar proportions of women (51%, n = 131; 51%, n = 1,282, respectively) and re-applicants (30%, n = 77; 30%, n = 764, respectively). A greater proportion of rural applicants reported a rural birth county 30% (n = 78) than urban applicants 4% (n = 96). Compared to rural applicants, a larger proportion of urban applicants were in the highest quintile of MCAT scores (12%, n = 30 vs. 17%, n = 426).

## Discussion

Based on the data from this study, our model analyzing rural pipeline programs demonstrated successful efforts to recruit rural medical students. We demonstrated that rural applicants are proportional to the potential applicant pool in the state’s rural counties and are admitted in proportion to their peers from nonrural backgrounds. Overall, 9.2% of applicants from rural backgrounds are close to parity with the potential rural applicant pool, which is 10.9% of NC’s population of college-educated 18-34-year-olds living in a rural county.

The importance of pipeline programs for rural students has been shown to be helpful in recruitment efforts that alter their habitus [9,16]. UNC SOM Office of Rural Initiatives [20] supports students during medical school, providing innovative pathway programs into medical school. The Office of Rural Initiatives conducts county-wide outreach programs with high school students, similar to efforts undertaken in Kentucky, Nebraska, New Mexico, and Wisconsin [21]. The UNC SOM has also worked to expand the spots in UNC’s Science Enrichment Preparation Program and the Medical Education Development Program for students from rural NC.

Implications from this study have added to the body of evidence confirming the results of prior studies that rural applicants are equally likely or more likely to be admitted [6,15,22-24] but emphasize the need for more effective pathway programs encouraging more applicants from rural backgrounds. We defined success as the proportion of applicants from rural backgrounds aligning with the potential rural applicant pool. However, like many of our peer institutions, innovative incentives are needed to increase the pool of physicians returning to rural communities.

Interest in health careers begins at age 15 or younger and close associates stimulate their aspirations, with teachers being the most influential [13]. Although the theory of habitus explains why rural students may never pursue higher education [16], pipeline programs extending into high schools may alter students’ habitus [25]. The UNC SOM and other medical schools are building these pipelines [21,23]. For example, the Family Medicine Summer Academy is a three-day immersive experience for high school students from rural and underserved areas of the state.

We must also consider recruiting urban students to build the rural physician workforce. Relying only on students from rural backgrounds may be insufficient to meet the significant workforce needs in rural communities. UNC training medical students in high functioning rural teaching practices can role model the rewards of working in rural communities for students not from rural backgrounds [26]. Immersion programs help students envision careers in rural settings. For example, UNC SOM’s Fully Integrated Readiness for Service program [27] and the University of Washington’s TRUST program [28] allow students to graduate early and begin training in a primary care program at their institution. Medical students who go on to residency training in the state have a much higher probability of remaining in practice after graduation [29].

Limitations

Home address county data may not accurately capture the rural identity of UNC SOM applicants. High school county is a better predictor of rural affinity than birth or home address county but was not available [7,30]. Rurality is a continuum [31], and the “rural” definition used in this analysis may be more restrictive than the one used by the admissions committee. UNC SOM has several rural-focused medical education initiatives and has noted in multiple public fora that the school seeks applicants with “a rural heart” [32-34], suggesting that there is also a qualitative notion of rurality being considered by the committee that we were unable to assess in the dataset used for this study.

Another limitation is that this study investigates a single institution. However, in addition to describing our recruitment efforts, we have also cited several other programs in states such as Kentucky, Nebraska, New Mexico, Oklahoma, Washington, and Wisconsin.

## Conclusions

It is important for schools to examine their recruitment and admissions processes to regularly collect and monitor tracking data for students by county of origin. This data will ensure that admitted students reflect the applicant pool from rural communities. Although the results of this study focus on medical school admissions, future work will track medical students from rural counties to determine if they return to rural or underserved areas to practice after completing residency training. Using our model of analysis, we discovered our recruitment efforts have been successful. Implementing innovative pathway programs reaching back to high school as well as curricular innovations to attract students who are not from rural backgrounds can increase the potential applicant pool. Our model of innovation and analysis can be applied to improving the candidate pool for other marginalized and underrepresented communities.
